# Selective and competitive inhibition of kynurenine aminotransferase 2 by glycyrrhizic acid and its analogues

**DOI:** 10.1038/s41598-019-46666-y

**Published:** 2019-07-15

**Authors:** Yukihiro Yoshida, Hidetsugu Fujigaki, Koichi Kato, Kyoka Yamazaki, Suwako Fujigaki, Kazuo Kunisawa, Yasuko Yamamoto, Akihiro Mouri, Akifumi Oda, Toshitaka Nabeshima, Kuniaki Saito

**Affiliations:** 1 Department of Disease Control and Prevention, Fujita Health University Graduate School of Health Sciences, Aichi, 470-1192 Japan; 2 College of Pharmacy, Kinjo Gakuin University, Aichi, 463-8521 Japan; 3 Faculty of Pharmacy, Meijo University, Aichi, 468-8503 Japan; 4 Advanced Diagnostic System Research Laboratory, Fujita Health University Graduate School of Health Sciences, Aichi, 470-1192 Japan; 5 Department of Regulatory Science, Fujita Health University Graduate School of Health Sciences, Aichi, 470-1192 Japan; 6 Japanese Drug Organization of Appropriate Use and Research, Aichi, 468-0069 Japan; 7 Human Health Sciences, Graduate School of Medicine and Faculty of Medicine, Kyoto University, Kyoto, 606-8507 Japan

**Keywords:** Enzymes, Computational biology and bioinformatics

## Abstract

The enzyme kynurenine aminotransferase (KAT) catalyses the conversion of kynurenine (KYN) to kynurenic acid (KYNA). Although the isozymes KAT1–4 have been identified, KYNA is mainly produced by KAT2 in brain tissues. KNYA is an antagonist of N-methyl-D-aspartate and α-7-nicotinic acetylcholine receptors, and accumulation of KYNA in the brain has been associated with the pathology of schizophrenia. Therefore, KAT2 could be exploited as a therapeutic target for the management of schizophrenia. Although currently available KAT2 inhibitors irreversibly bind to pyridoxal 5′-phosphate (PLP), inhibition via this mechanism may cause adverse side effects because of the presence of other PLP-dependent enzymes. Therefore, we identified novel selective KAT2 inhibitors by screening approximately 13,000 molecules. Among these, glycyrrhizic acid (GL) and its analogues, glycyrrhetinic acid (GA) and carbenoxolone (CBX), were identified as KAT2 inhibitors. These compounds were highly selective for KAT2 and competed with its substrate KYN, but had no effects on the other 3 KAT isozymes. Furthermore, we demonstrated that in complex structures that were predicted in docking calculations, GL, GA and CBX were located on the same surface as the aromatic ring of KYN. These results indicate that GL and its analogues are highly selective and competitive inhibitors of KAT2.

## Introduction

**Figure 1:**
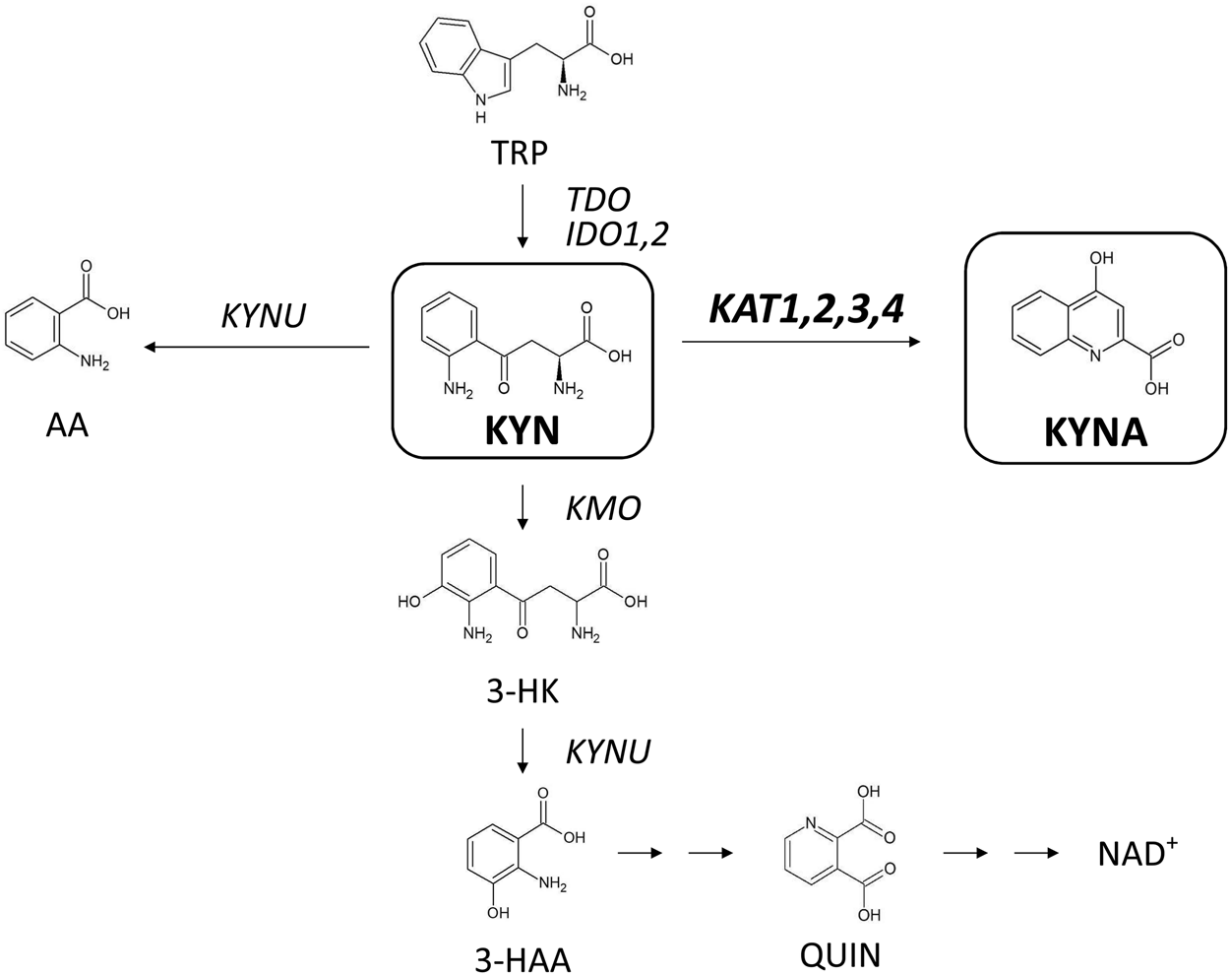
Schematic overview of the tryptophan-kynurenine pathway. Kynurenic acid (KYNA) is formed from kynurenine (KYN) by kynurenine aminotransferase (KAT) enzymes. TRP, tryptophan; AA, anthranilic acid; 3-HK, 3-hydroxy kynurenine; 3-HAA, 3-hydroxy anthranilic acid; QUIN, quinolinic acid; TDO, tryptophan 2,3-dioxygenase; IDO1, indoleamine 2,3-dioxygenase 1; IDO2, indoleamine 2,3-dioxygenase 2; KYNU, kynureninase; KMO, kynurenine 3-monooxygenase; NAD^+^, nicotinamide adenine dinucleotide.

Dietary tryptophan is predominantly metabolised via the kynurenine pathway^1^ (Fig. 1), which produces neuroactive metabolites such as kynurenic acid (KYNA). KYNA reportedly acts as an antagonist of N-methyl-D-aspartate receptors^2,3^ and the α-7-nicotinic acetylcholine receptor (α7nAChR)^4,5^. Furthermore, previous studies in animal models show that elevated KYNA levels in the brain impair cognitive function^6^, spatial working memory^7^ and auditory sensory gating^8^ in rats. Abnormally high KYNA levels have been observed in brain tissues and cerebral spinal fluid (CSF) from patients with schizophrenia^9–11^. Therefore, physiological control of brain KYNA levels is likely critical to the prevention of these conditions^1^.

KYNA is produced from kynurenine (KYN) by kynurenine aminotransferase (KAT) (Fig. 1). KAT enzymes require pyridoxal 5′-phosphate (PLP) coenzymes, and four KAT isoforms (KAT1–4) have been identified. In the brain, KYNA is predominantly generated by KAT2, which is expressed in astrocytes^12–14^. Accordingly, KAT2 plays essential roles in regulating KYNA levels in the brain.

Currently, various KAT2 inhibitors, including PF-04859989^15^, BFF-122^16^, BFF-816^17,18^ and S-ESBA^19^, have been shown to decrease KYNA levels in the brain. In animal models, these KAT2 inhibitors improve cognitive function by releasing dopamine, acetylcholine and glutamate^20–23^. PF-04859989 and BFF-122 also act as KAT2 inhibitors by irreversibly binding to the PLP cofactor^15,16^. However, because over 300 PLP-dependent enzymes and proteins have been identified, irreversible binding to PLP may cause side effects. For example, carbidopa is used for the treatment of Parkinson’s disease, but causes adverse effects due to irreversible binding to PLP^24,25^.

**Figure 2:**
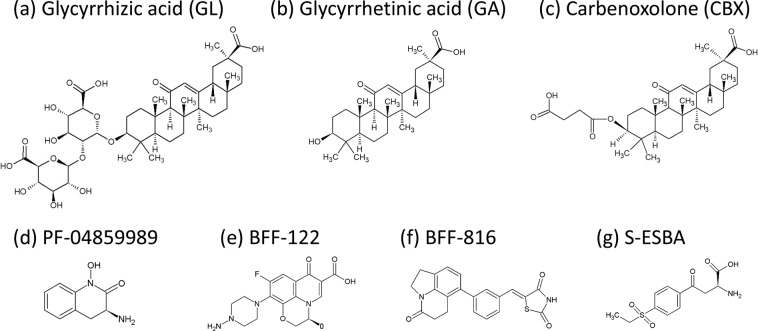
Chemical structures of (**a**) glycyrrhizic acid (GL), (**b**) glycyrrhetinic acid (GA) and (**c**) carbenoxolone (CBX). Chemical structures of the known KAT2 inhibitors (**d**) PF-04859989, (**e**) BFF-122, (**f**) BFF-816 and (**g**) S-ESBA are also presented.

In this study, we identified novel KAT2 inhibitors with different inhibitory mechanisms using a microplate fluorescence assay for kynurenine aminotransferase as reported previously^26^ with minor modifications. We then screened for KAT2 inhibitors from approximately 13,000 compounds using a high-throughput screening assay. These experiments identified approximately 20 candidate KAT2 inhibitors (data not shown), including glycyrrhizic acid (GL) and its analogues, glycyrrhetinic acid (GA) and carbenoxolone (CBX) (Fig. 2). The basic structures of these three compounds are similar. GL comprises GA and two glucuronic acid molecules^27,28^, whereas CBX is a derivative of GA^29^. GL is also the main component of liquorice, which is a component of the traditional Japanese medicine yokukansan^30^. Previous reports show that yokukansan alleviates psychological symptoms of schizophrenia^31,32^ and the behavioural and psychological symptoms of dementia in patients with Alzheimer’s disease and Lewy bodies^33^ and improves cognitive function in patients with senile dementia^34,35^. Thus, GL, GA and CBX may be KAT2 inhibitors with efficacy in treatment of mental disorders.

## Results

### Inhibitory activity of GL and its analogues

**Figure 3:**
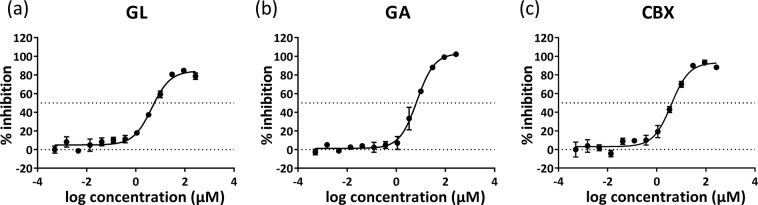
Dose dependent inhibitory activities of GL and its analogues on human KAT2. Half inhibitory concentrations (IC_50_) of (**a**) GL, (**b**) GA and (**c**) CBX were analysed using nonlinear curve fitting. All experiments were performed in triplicate and data are presented as means ± standard errors of the mean (SEM).

**Figure 4:**
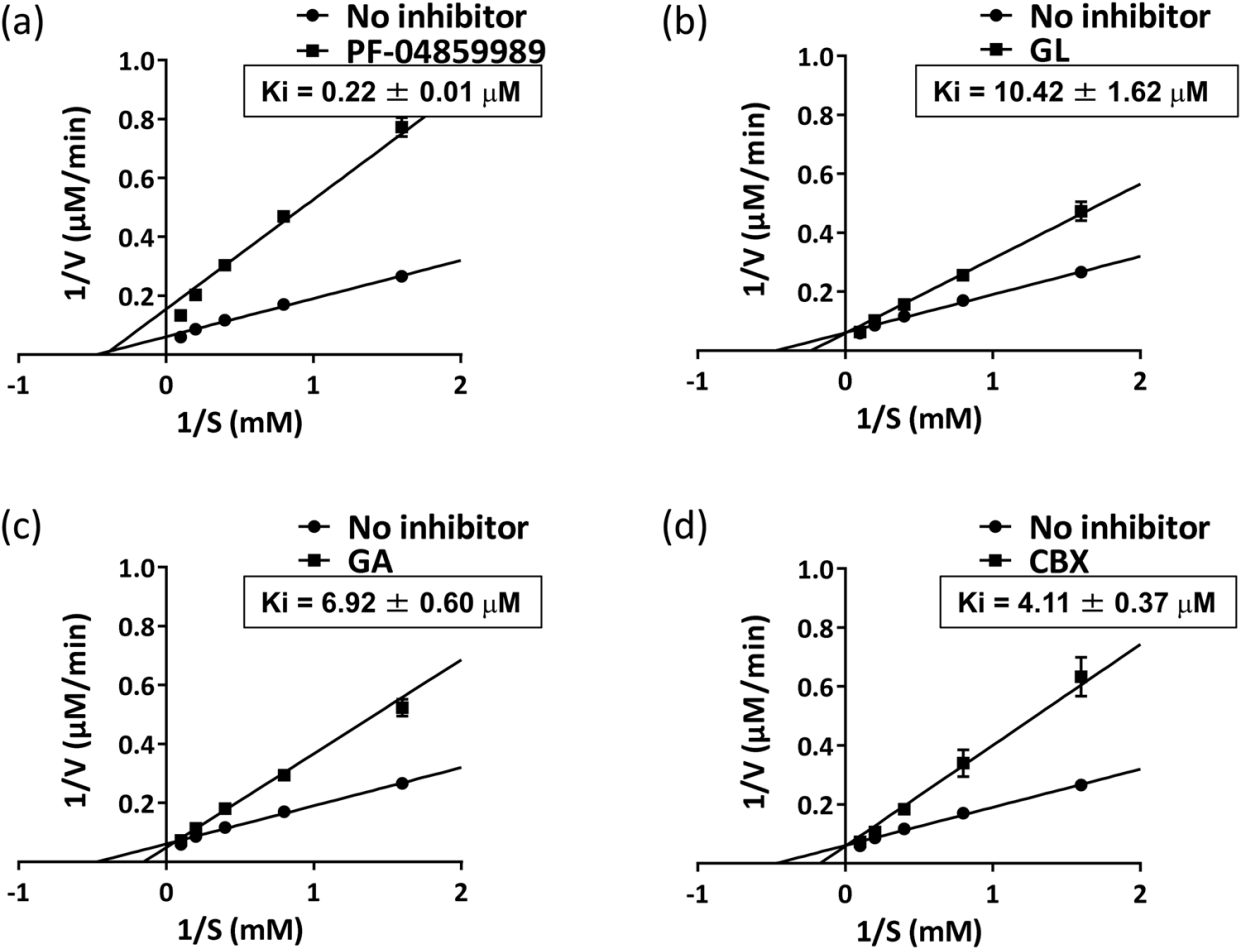
Lineweaver–Burk plots of the inhibitory kinetics of PF-04859989, GL and its analogues towards KAT2. Inhibition kinetics of (**a**) PF-04859989, (**b**) GL, (**c**) GA and (**d**) CBX were evaluated using Lineweaver–Burk analyses. All experiments were performed in triplicate and data are presented as means ± SEM. Analyses were performed in the presence of varying concentrations of KYN and KAT2 inhibitor. Inhibitor concentrations: (a) PF-04859989, 300 nM; (b) GL, 4.5 μM; (c) GA, 6.0 μM; (d) CBX, 3.5 μM; Ki values were calculated using the global fit formula (GraphPad Prism v6.07).

To evaluate the inhibitory activities of GL, GA and CBX against KAT2, we measured half-maximal inhibitory concentrations (IC_50_) and estimated inhibition constants (Ki). GL (IC_50_, 4.51 ± 0.20 μM; Ki, 10.42 ± 1.62 μM), GA (IC_50_, 6.96 ± 0.37 μM; Ki, 6.92 ± 0.60 μM) and CBX (IC_50_, 3.90 ± 0.37 μM; Ki, 4.11 ± 0.37 μM) showed high inhibitory activity against human KAT2 (Figs 3 and 4). In investigations of the selectivity of GL, GA and CBX for KAT isoforms, all three compounds showed inhibitory activity against mouse KAT2, but none inhibited the other 3 KAT isozymes (Table 1), indicating selectivity for KAT2.

**Table 1:** Selective inhibition of KAT isoenzyme by glycyrrhizic acid (GL), glycyrrhetinic acid (GA) and carbenoxolone (CBX).

Enzymes	IC_50_ (μM)		
	Glycyrrhizic acid	Glycyrrhetinic acid	Carbenoxolone
human KAT2	4.51 ± 0.20	6.96 ± 0.37	3.90 ± 0.37
mouse KAT2	86.21 ± 1.94	56.84 ± 1.45	19.83 ± 0.34
human KAT1	>500	>500	>500
human KAT3	>500	236.23 ± 16.49	>500
human KAT4	>500	>500	>500

Data are presented as means ± standard errors of the mean (SEM) from three separate experiments.

### Inhibitory mechanisms of the compounds against human KAT2

To investigate inhibitory mechanisms of GL, GA and CBX, we measured KAT2 activity in the presence of varying KYN concentrations and inhibitory compounds at close to their IC_50_ (GL, 4.5 μM; GA, 6.0 μM; CBX, 3.5 μM). Additionally, we then compared inhibitory properties of these compounds with those of the KAT2 inhibitor PF-04859989, which binds PLP irreversibly. PF-04859989 was used at its IC_50_ value of 300 nM, as determined in our assays. Because PF-04859989 binds to PLP, maximum reaction rates (V_max_) varied but the Michaelis–Menten constant (K_m_) did not change (Fig. 4a). In contrast, K_m_ values for GL, GA and CBX changed with substrate concentrations, whereas respective V_max_ values did not vary under these conditions (Fig. 4b–d). These results showed that GL, GA and CBX do not bind PLP, but act as competitive inhibitors of KYN.

### Computational docking of GL and its analogues to KAT2

**Figure 5:**
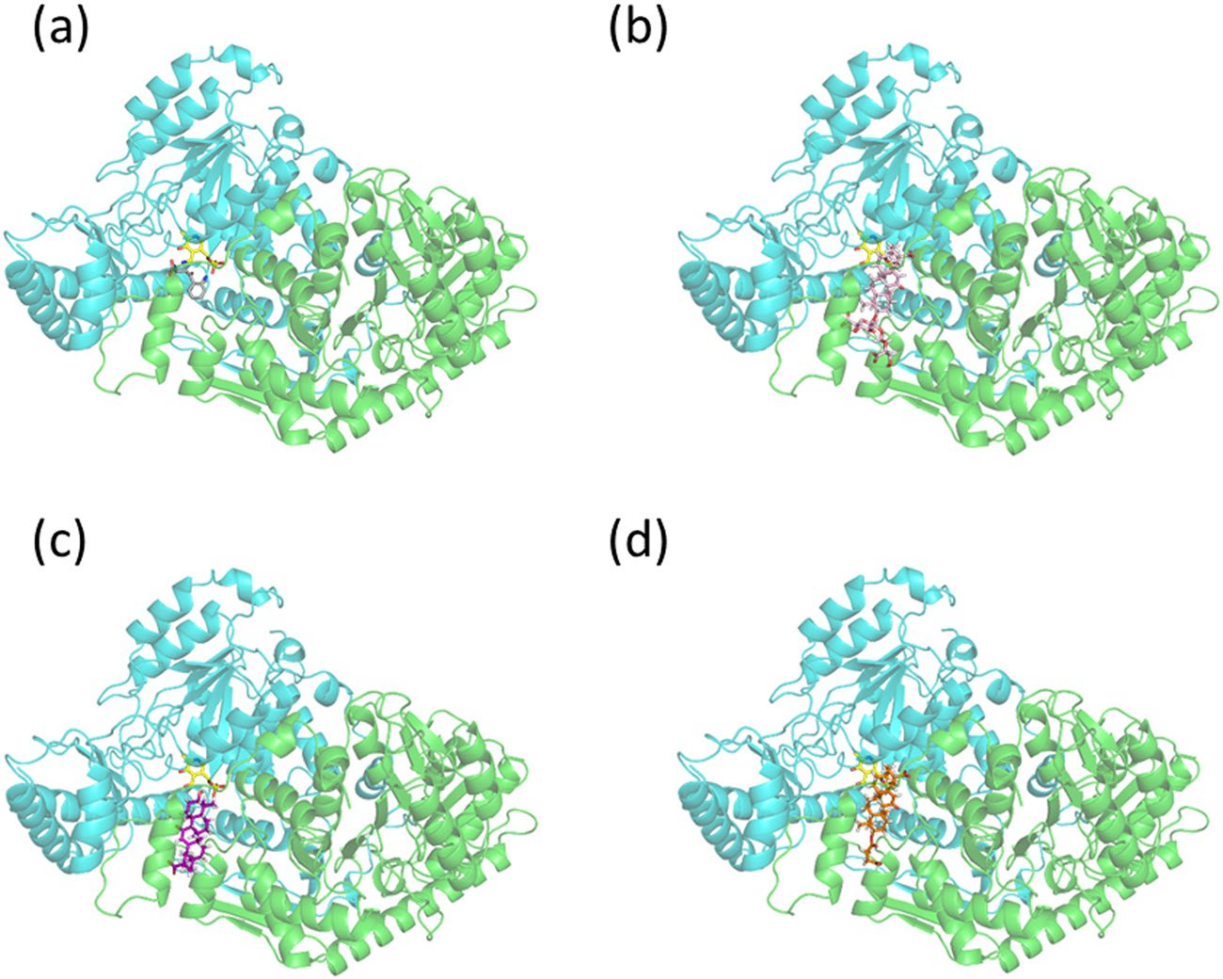
The complex structures of KAT2 with GL and its analogues. (**a**) Crystal structure of KAT2-KYN (PDB ID, 2R2N) and (**b**) predicted structures of KAT2-GL, (**c**) KAT2-GA and (**d**) KAT2-CBX. In the homodimer of KAT2, the two subunits A and B are shown in green and cyan, respectively. KYN is presented in grey, GL is shown in pink, GA is shown in purple, CBX is shown in orange, PMP and PLP are shown in yellow.

**Figure 6:**
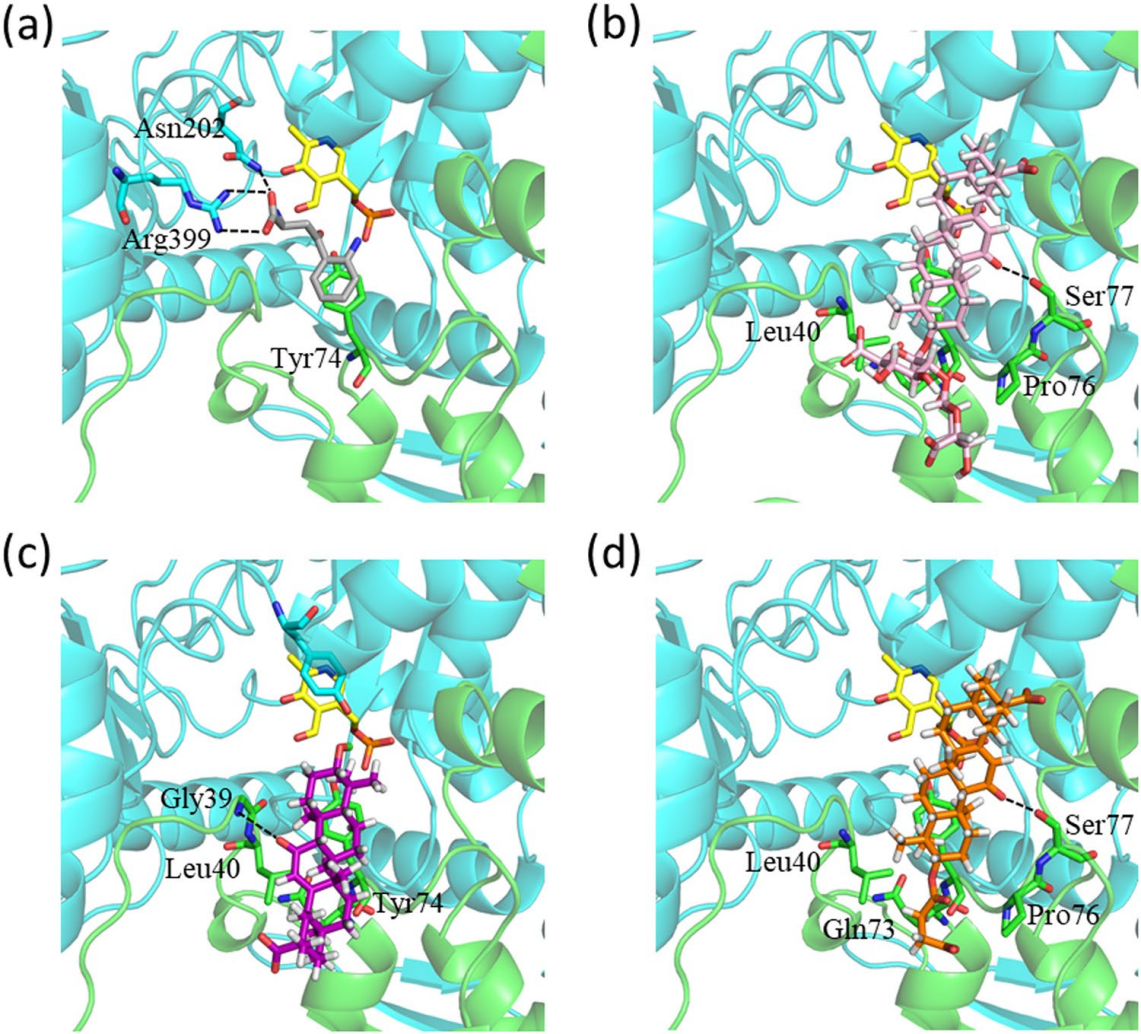
Docking models of GL and its analogues with the ligand-binding pocket of KAT2. Ligand binding pockets in (**a**) the crystal structure of 2R2N; (**b**) predicted structures for GL, (**c**) GA and (**d**) CBX are illustrated. Colour coordinates are the same as in Fig. 5. The residues that are involved in interactions with ligands are shown in stick models. Atom colour; H (white), O (red) and N (blue). Dotted lines represent hydrogen bonds.

**Figure 7:**
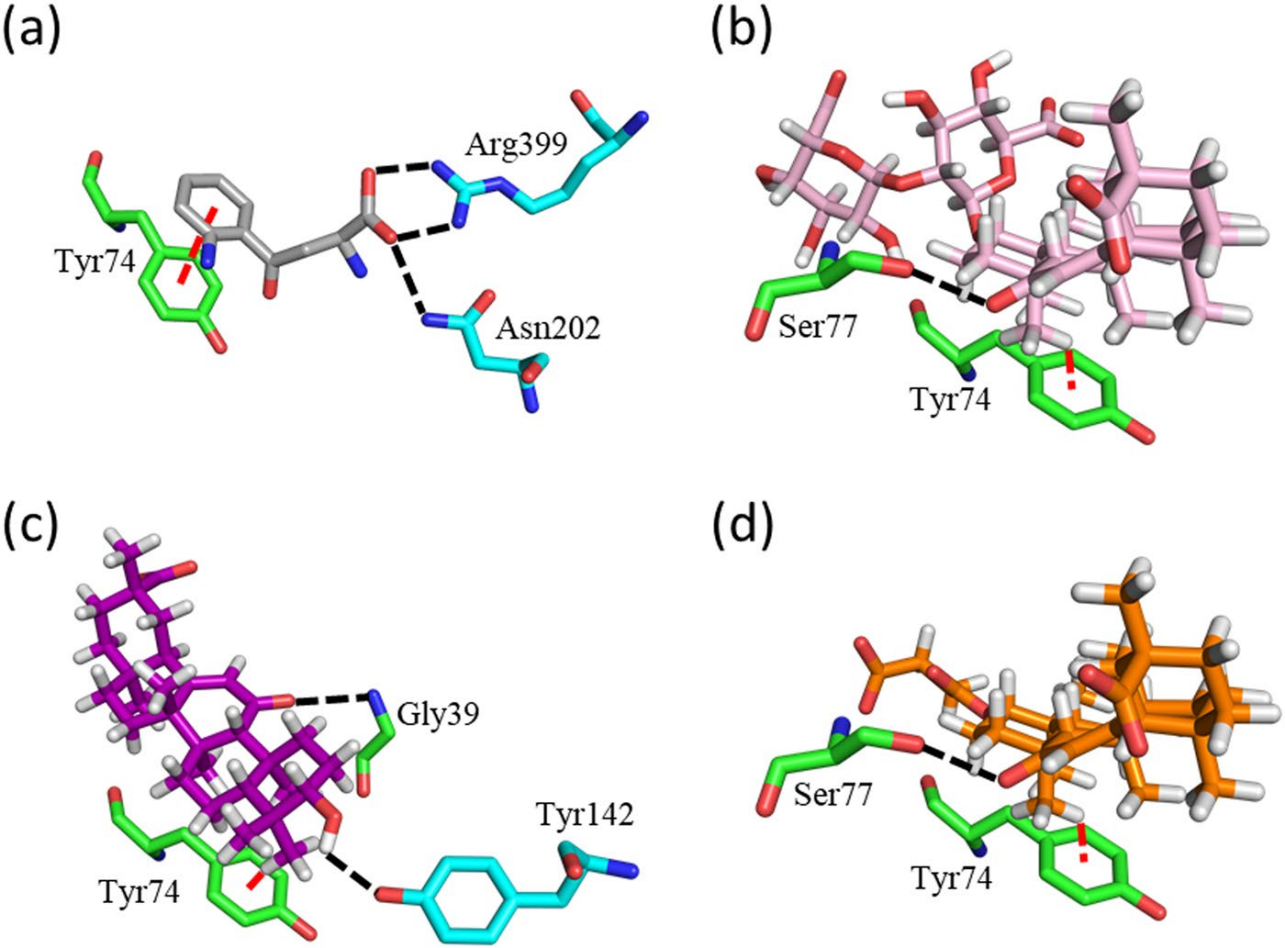
Key KAT2 residues for binding of GL and its analogues. Residues that are involved in hydrogen-bonding formations and stacking interactions in (**a**) the crystal structure of 2R2N, (**b**) predicted structures for GL, (**c**) GA and (**d**) CBX are illustrated. The colour coordinates are as in Fig. 5. Residues are shown with stick models with the atoms H (white), O (red) and N (blue). Black dotted lines represent hydrogen bonds. Red dotted lines show possible stacking interactions.

Three-dimensional structures of KAT2-ligand complexes were obtained using the docking calculations shown in Fig. 5. For comparison, we also present a crystal structure from the protein data bank (PDB; PDB ID, 2R2N) in Fig. 5a. All ligands were located in the ligand-binding pocket of two KAT2 molecules. To elucidate the interactions between KAT2 and these ligands, we magnified the structures of ligand-binding pockets (Fig. 6), and identified residues that are involved in hydrogen-bond formation and stacking, as shown in Fig. 7. In these figures, the subunits A and B are shown in green and cyan, respectively. In the crystal structure of 2R2N, KYN forms hydrogen bonds with Asn202(B) and Arg399(B) (Figs 6a and 7a), and the binding structure is stabilised by a π-π interactions between the aromatic ring of KYN and the Tyr74(A) side chain. Although GL formed a hydrogen bond with Ser77(A), no hydrogen bonds were observed between GL and Asn202(B) or Arg399(B) (Figs 6b and 7b). Yet CH-π stacking interactions with Tyr74(A) may stabilise the binding structure. The hydrophobic moiety of GL was located on hydrophobic surfaces comprising Leu40(A), Tyr74(A), Pro76(A) and Gln73(A) and the carbohydrate chain of GL was located outside of the ligand-binding pocket. Similarly, the hydrophobic moiety of GA was located on the hydrophobic surface of Leu40(A), Tyr74(A), Pro76(A) and Gln73(A) (Fig. 6c), and the binding structure of GA favours stabilisation by CH-π stacking interactions with Tyr74(A) (Fig. 7c). Although GA formed no hydrogen bonds with Asn202(B), Arg399(B) or Ser77(A), hydrogen bonds with Gly39(A) and Tyr142(B) were observed. The docking pose of CBX, which had a similar IC_50_ value to that of GL, was also similar to that of GL (Figs 6d and 7d). Moreover, the residues required for stabilisation of ligand binding by hydrophobic effects and stacking interactions were conserved among GL and its analogues (Table 2). In particular, Tyr74 is considered one of the most important residues for ligand binding.

**Table 2:** Important KAT2 binding residues for glycyrrhizic acid (GL), glycyrrhetinic acid (GA) and carbenoxolone (CBX).

Interactions	KYN	GL	GA	CBX
Hydrogen bond	Asn202(B), Arg399(B)	Ser77(A)	Gly39(A) Tyr142(B)	Ser77(A)
Hydrophobic effects	Leu40(A) Tyr74(A)	Leu40(A) Tyr74(A) Pro76(A) Gln73(A)	Leu40(A) Tyr74(A) Pro76(A) Gln73(A)	Leu40(A) Tyr74(A) Pro76(A) Gln73(A)
Stacking	Tyr74(A)	Tyr74(A)	Tyr74(A)	Tyr74(A)

## Discussion

In this study, we examined the inhibitory effects of GL, GA and CBX on KAT2 using recombinant proteins. Our data show that GL, GA and CBX are strong KAT2 inhibitors and compete with the substrate KYN.

Previous studies have indicated that increased KYNA concentrations in the brain impair cognitive function^6^, spatial working memory^7^ and auditory sensory gating^8^ in rats. Moreover, abnormal increases in KYNA have been observed in brain tissues and CSF from patients with schizophrenia^9–11^. Because KYNA production in the brain is predominantly catalysed by KAT2^12–14^, KAT2 inhibitors effectively control KYNA excesses in the brain, leading to improved cognitive function and spatial memory in rats^23,36^. Moreover, KAT2-knockout mice displayed improvements in cognitive function, as observed with KAT2 inhibitors^37^.

Several KAT2 inhibitors are currently available. Among these, PF-04859989 and BFF-122 irreversibly bind PLP, which is a cofactor of all KAT isoforms^15,16^. Irreversible binding to PLP may lead to undesirable inhibition of over 300 PLP-dependent enzymes and proteins. S-ESBA and NS-1502 are KAT2 inhibitors that show selective and reversible inhibition of KAT2^38,39^. But, reported IC_50_ values for these compounds with human KAT2 are very high (S-ESBA, approximately 1000 μM; NS-1502, 315 μM)^38,39^. In studies with the recently developed KAT2 inhibitor BFF-816^17,18^, oral administration prevented increases in KYNA in the brain after systemic kynurenine injections and attenuated the resulting glutamate release in the rat prefrontal cortex^18^. The mechanisms of action (competitive or non-competitive) and selectivity of BFF816 for KAT isoforms remains unclear.

In this study, we identified novel KAT2 inhibitors with strong inhibitory effects. The IC_50_ values of these compounds (GL, GA and CBX) were low μM range on human KAT2 (Table 1). Although these IC_50_ values were determined at the basic conditions (pH9.5) to obtain the optimal fluorescence^26^, at the physiological conditions (pH 7.4), we confirmed that the IC_50_ values of GL, GA and CBX on human KAT2 were almost the same as those of pH9.5 (7.93 ± 0.17 μM, 9.87 ± 0.32 μM, and 3.34 ± 0.25 μM, respectively). We also showed that these compounds are highly selective for KAT2 among KAT isozymes and compete with its substrate KYN. Furthermore, we predicted complex structures using docking calculations and located GL, GA and CBX on the same surface as the aromatic ring of KYN. These docking structures suggest that the high inhibitory activities of GL and its analogues reflect larger hydrophobic interfaces with KAT2 than with KAT2-KYN. In agreement, the hydrophobic effects of Leu40 and Tyr74 are common among KAT2 inhibitors that are described in previous studies^15,16,40,41^. Furthermore, Gly39 forms a hydrogen bond with GA and with BFF-122^16^ and the hydrogen bond between the inhibitor and Tyr142 of the KAT2-GA complex was also formed in the complex of KAT2 and NS-15024. In contrast, the hydrogen bond with Ser77 is unique to docking structures of GL and CBX. Yet the carbohydrate chain of GL, which is located outside of the ligand-binding pocket, is not likely to affect the affinity for KAT2. Because these inhibitors merely alter PLP binding, the risks of side effects may be minimised in comparison with those of PF-04859989 and BFF-122, which inhibit KAT2 by forming covalent adducts with PLP. In addition, compared with S-ESBA and NS-1502, which exhibit reversible inhibitory effects, GL, GA and CBX have potent inhibitory activities. Therefore, we successfully identified three new KAT2 inhibitors that are superior to other KAT2 inhibitors.

The present experiments show that the inhibitory effects of GL, GA and CBX on KAT2 are less potent in mice than in humans (Table 1). These differences may arise from differences in conserved residues among species. Accordingly, in the sequence alignments of mouse, rat and human KAT2 reported by Pellicciari *et al*., sequence variants in human and rat KAT2 were associated with relative inhibitory activities of S-ESBA^38^. Our computational docking study shows that the hydrophobic effects of Leu40 in human KAT2 are important for its interactions with GL and its analogues (Table 2). Among residues that participate in binding of human KAT2 to GL and its analogues, Leu40 is substituted to Ser in mouse KAT2. We speculate that this difference undermines the inhibitory effects of GL and its analogues on mouse KAT2.

We also demonstrated that among the four KAT isoenzymes (KAT1–4), GL, GA and CBX selectively inhibit KAT2 (Table 1). Because KYNA is predominantly generated by KAT2 in brain tissues^12–14^, specific inhibitors of KAT2 are eagerly awaited. Rossi, F. *et al*. solved the crystal structures of human KAT1 and KAT2, and showed important structural differences that could be exploited in the rational design of highly selective KAT2 inhibitors^42–45^. In particular, human KAT1 and KAT2 share 15% overall sequence identity, and structural analyses of their active sites revealed striking differences in substrate binding pockets^42,45^ (for review see^43,44^). We also demonstrated that hydrophobic interactions between Leu40(A) and Tyr74(A) with GL and its analogues are similar to those of the KAT2 inhibitors reported in previous studies. But we suggest that Pro76(A) and Gln73(A) are additional unique residues that interact with GL and its analogues (Table 2). These residues may be involved in the selective inhibitory effects of GL and its analogues for human KAT2. Although the mechanisms through which GL and its analogues specifically inhibit KAT2 remain unknown, these structural differences between KAT isozymes and the unique residues that interact with GL and its analogues are likely mediators of specificity. Hence, the present observations can be used to inform the rational design of specific and competitive KAT2 inhibitors.

KAT2 inhibitors with therapeutic potential for psychiatric disorders must pass through the blood brain barrier (BBB). CBX failed to pass through the BBB in previous reports^46^, precluding further consideration. In contrast, GL, which is the main constituent of liquorice and comprises a molecule of GA and two glucuronic acid molecules^27,28^, is metabolised to GA by microbiota in the intestine following oral administration, and GA is then absorbed into the bloodstream^47,48^ and through the BBB^30^. Hence, further studies are warranted to clarify the KAT2 inhibitory effects of GL in animal models of psychiatric diseases.

Liquorice is an ingredient of yokukansan, which is a traditional Japanese Kampo medicine. Yokukansan is effective as a treatment for patients with symptoms of schizophrenia^31,32^ and in patients with behavioural or psychological symptoms of dementia due to Alzheimer disease and Lewy bodies^33–35^. Yet, yokukansan has multiple ingredients and it is not clear which of these are responsible for the therapeutic effects. Yokukansan reportedly inhibits glutamate release in zinc-deficient rats^49^ and exerts neuroprotective effects by accommodating dysfuntional glutamate transporters in astrocytes^50^. The inhibitory effects of yokukansan on KAT2 have not, however, been clarified. Further studies are required to evaluate the involvement of KAT2 inhibition in the neuroprotective and therapeutic effects of yokukansan in patients with psychiatric disorders.

As a pharmacological agent, GL has anti-inflammatory^51,52^ and antiviral activities^53^ and inhibits hepatic fibrosis^54^ and tumour growth^55^. Following improvements in solubility, Glycyrrhizin salts, such as diammonium glycyrrhizinate and dipotassium glycyrrhizinate, have anti-inflammatory effects that are similar to those of GL^56,57^. Furthermore, drugs containing GL, such as stronger neo-minophagen C and glycyron tablets (Minophagen Pharmaceutical, Tokyo, Japan), are commercially available and have been used to treat chronic liver disease^58,59^. Thus, the use of GL as a KAT2 inhibitor for the treatment of schizophrenia represents a drug repositioning process through which new therapeutic uses for existing drugs are discovered.

In conclusion, we demonstrate that GL, GA and CBX are inhibitors of KAT2. These inhibitors were selective and potent, and operated through mechanisms of substrate competition. Thus, our findings suggest that drugs containing GL are excellent candidates for drug repositioning in the treatment of schizophrenia.

## Methods

### Chemicals

GL, GL dipotassium salt, GA and dimethyl sulfoxide (DMSO) were purchased from FUJIFILM Wako Pure Chemical, Ltd. (Osaka, Japan). CBX was purchased from Abcam (UK). All compounds were dissolved in DMSO.

### Enzyme production

All methods were carried out in accordance with relevant guidelines and regulations. The protocol for all animal experiments was approved by the Institutional Animal Care and Use Committee of Fujita Health University. Human *KAT1*, *KAT2* and *KAT4* cDNAs were synthesised from human blood peripheral leukocytes total RNA (TaKaRa, Japan) using a ReverTra Ace Kit (Toyobo, Osaka, Japan). Mouse *KAT2* cDNA was synthesised from total RNA that was extracted from whole brains of mice using a ReverTra Ace Kit. All cDNAs were amplified using polymerase chain reactions with specific primers.

Amplified cDNAs were cloned into the pFastBac HTC vector (Invitrogen, Carlsbad, CA, USA), which was transformed into *Escherichia coli* DH5α cells. The pFastBac HTC vector containing the target gene was transformed into *E. coli* DH10Bac cells and a baculovirus transfer vector was transfected into insect Sf9 cells. Recombinant enzymes were expressed by infection of Sf9 cells with a high-titre baculovirus.

Sf9 cells were pelleted by centrifugation and were then dissolved in 50 mM phosphate buffer (pH 8.0) containing 300 mM NaCl and 10 mM imidazole. After sonication, cell lysates were centrifuged at 10,000 × *g* for 20 min at 4 °C, and recombinant enzymes in supernatants were added to pre-equilibrated Ni-NTA resin (Qiagen). Enzyme/resin complexes were transferred to columns, were washed with buffer containing 300 mM NaCl and 20 mM imidazole in 50 mM phosphate buffer (pH 8.0), and recombinant enzymes were eluted with buffer containing 300 mM NaCl, 250 mM imidazole and 50 mM phosphate (pH 8.0). Enzyme fractions were pooled based on purity, as determined using sodium dodecyl sulfate polyacrylamide gel electrophoresis, and were then desalted using PD-10 columns (GE Healthcare, UK). Recombinant human KAT3 was purchased from OriGene Technologies, Inc. (USA).

### High-throughput screening assays for inhibitors of human KAT2

High-throughput screening assays for inhibitors of human KAT2 were conducted using a microplate fluorescence assay for kynurenine aminotransferase^26^ with minor modifications. In these assays, KAT2 enzyme activities were measured in black 384-well untreated plates. The human KAT2 reaction mixture (20 μL) contained 10 ng/μL recombinant human KAT2, 1 mM L-KYN, 1 mM α-ketoglutaric acid, 500 μM PLP, 0.005% Tween 20 and 150 mM AMP buffer (pH 9.5), and was added to 384-well plates containing compounds using a Multidrop dispenser (Thermo Fisher Scientific, USA). The compound library comprised about 13,000 compounds from the Drug Discovery Initiative at the University of Tokyo. The compound library includes about 9,600 diverse compounds for pilot screening and about 3,400 known bioactive compounds. All compounds were dissolved and diluted in DMSO to a final concentration of 10 μM. Reaction mixtures were incubated for 2 h at room temperature, and 20 μL aliquots of 300 mM zinc acetate (pH 5.5) were then added directly using a Multidrop dispenser. Fluorescence intensities of KYNA were measured using an ARVO X Multi Label Reader (PerkinElmer, USA) at an excitation wavelength of 340 nm and an emission wavelength of 460 nm. Assay quality was validated by calculating signal–background and Z’ factor. These assays identified approximately 20 candidate KAT2 inhibitors with potent inhibitory activity from about 13,000 compounds. Candidate compounds were validated in quadruplicate KAT2 enzyme activity assays.

### Enzyme inhibition and kinetics assays

Inhibitory activities of the identified compounds against KAT1 and KAT2 were measured using the enzyme activity assays described above. KAT1 reaction mixtures (20 μL) contained 10 ng/μL recombinant human KAT1, 1 mM L-KYN, 1 mM sodium pyruvate, 100 μM PLP, 0.005% Tween 20 and inhibitors at various concentrations in 150 mM 2-amino-2-methyl-1-propanol (AMP) buffer (pH 7.4 or 9.5). Human and mouse KAT2 reaction mixtures (20 μL) contained 10 ng/μL recombinant human or mouse KAT2, 1 mM L-KYN, 1 mM α-ketoglutaric acid, 100 μM PLP, 0.005% Tween 20 and inhibitors at various concentrations in 150 mM AMP buffer (pH 9.5). Following the addition of 20 μL aliquots of 300 mM zinc acetate, fluorescence intensities of KYNA were measured using an ARVO X Multi Label Reader (PerkinElmer, USA).

KAT3 and KAT4 enzyme activities were measured using high performance liquid chromatography (HPLC) analyses of reaction products. Briefly, KAT3 reaction mixtures (50 μL) contained 10 ng/μL recombinant human KAT3, 1 mM L-KYN, 1 mM sodium pyruvate, 100 μM PLP, 0.005% Tween 20 and inhibitors at various concentrations in 150 mM AMP buffer (pH 9.5). KAT4 reaction mixtures (50 μL) contained recombinant human KAT4, 1 mM L-KYN, 1 mM α-ketoglutaric acid, 100 μM PLP, 0.005% Tween 20 and inhibitors at various concentrations in 100 mM Tris buffer (pH 7.5). Reaction mixtures were incubated for 1 h at 37 °C and reactions were then stopped by adding 3% perchloric acid at a ratio of 1:1. KYNA contents of reaction mixtures were then determined using HPLC (Shimadzu, Kyoto, Japan) following isocratic elution from a reverse-phase column (TSKgel ODS-100V, 3 μm, 4.6 mm [ID] × 150 mm [L]; Tosoh, Tokyo, Japan) using a mobile phase containing 10 mM sodium acetate with 2% acetonitrile (pH adjusted to 4.5) at flow rate of 0.8 mL/min. KYNA was detected using a fluorescence detector (RF-20Axs) at an excitation wavelength of 344 nm and an emission wavelength of 380 nm.

To determine steady state kinetic parameters for the substrate L-KYN, 50 μL reaction mixtures containing 10 ng/μL recombinant human KAT2, 1 mM α-KG, 100 μM PLP, 0.005% Tween 20, L-KYN at concentrations of 10, 5, 2.5, 1.25, 0.62, 0.31 and 0.15 mM and GL, GA or CBX or PF-04859989 at 4.5, 6.0 and 3.5 μM and 300 nM, respectively. Following incubation for 30 min at 37 °C, reactions were stopped by adding 3% perchloric acid at a 1:1 ratio. KYNA concentrations were measured using the same HPLC method as described above. Double reciprocal plots were used to determine types of inhibition. Competitive inhibition constants (Ki) for GL, GA and CBX were calculated using the global fit formula (GraphPad Prism v6.07)^60,61^ as follows:

$${\rm{v}}={\rm{Vmax}}\ast [{\rm{S}}]/({{\rm{Km}}}_{{\rm{obs}}}+[{\rm{S}}])\,{\rm{and}}\,{{\rm{Km}}}_{{\rm{obs}}}={\rm{Km}}\times (1+[{\rm{I}}]/{\rm{Ki}}),$$

where v = initial velocity, Vmax = maximum velocity, [S] = substrate concentration, Km = Michaelis–Menten constant and [I] = inhibitor concentration. The Ki value of PF-04859989 was calculated using the global fit formula as follows:

$${{\rm{Vmax}}}_{{\rm{inh}}}={\rm{Vmax}}/(1+[{\rm{I}}]/{\rm{Ki}})\,{\rm{and}}\,{{\rm{Km}}}_{{\rm{obs}}}={{\rm{Vmax}}}_{{\rm{inh}}}\times [{\rm{S}}]/({\rm{Km}}+[{\rm{S}}]),$$

where Vmax_inh_ = maximum enzyme velocity for the concentration of inhibitor. IC_50_ values were determined by nonlinear curve fitting using GraphPad Prism v6.07. All statistical analyses were performed using GraphPad Prism v6.07.

### Docking calculations

Computational docking trials were performed using GOLD 5.6 software under default settings^62^. The three-dimensional (3D) structure of KAT2, which forms a homo-dimer^63,64^, was retrieved from the Protein Data Bank (PDB; PDB ID, 2R2N). Because this crystal structure includes the substrate KYN and the coenzyme analogue pyridoxamine 5′-phosphate (PMP), KYN was removed, and PLP was substituted for PMP before calculations. For this substitution, the PLP structure was optimised using Gaussian16 (Gaussian Inc., Wallingford CT, 2016) with B3LYP/6-31 + G(d,p). Missing hydrogen atoms in the PDB structure were computationally added using Hermes (https://www.ccdc.cam.ac.uk/). The centre of KYN in 2R2N was defined as the centre of the ligand-binding site, and the binding site radius was set at 10.0 Å. Ligand structures were optimised at the same calculation level as for PLP.
